# The role of vaccination route with an adenovirus-vectored vaccine in protection, viral control, and transmission in the SARS-CoV-2/K18-hACE2 mouse infection model

**DOI:** 10.3389/fimmu.2023.1188392

**Published:** 2023-08-16

**Authors:** Alexandria Dickson, Elizabeth Geerling, E. Taylor Stone, Mariah Hassert, Tara L. Steffen, Taneesh Makkena, Madeleine Smither, Katherine E. Schwetye, Jianfeng Zhang, Bertrand Georges, M. Scot Roberts, John J. Suschak, Amelia K. Pinto, James D. Brien

**Affiliations:** ^1^ Department of Molecular Microbiology and Immunology, Saint Louis University, St Louis, MO, United States; ^2^ Department of Pathology and Immunology, Washington University School of Medicine, St. Louis, MO, United States; ^3^ Altimmune Inc., Gaithersburg, MD, United States

**Keywords:** SARS-CoV-2, intranasal vaccination, intramuscular vaccination, virus transmission, vaccine efficacy, vaccine immunogenicity

## Abstract

**Introduction:**

Vaccination is the most effective mechanism to prevent severe COVID-19. However, breakthrough infections and subsequent transmission of SARS-CoV-2 remain a significant problem. Intranasal vaccination has the potential to be more effective in preventing disease and limiting transmission between individuals as it induces potent responses at mucosal sites.

**Methods:**

Utilizing a replication-deficient adenovirus serotype 5-vectored vaccine expressing the SARS-CoV-2 RBD (AdCOVID) in homozygous and heterozygous transgenic K18-hACE2, we investigated the impact of the route of administration on vaccine immunogenicity, SARS-CoV-2 transmission, and survival.

**Results:**

Mice vaccinated with AdCOVID via the intramuscular or intranasal route and subsequently challenged with SARS-CoV-2 showed that animals vaccinated intranasally had improved cellular and mucosal antibody responses. Additionally, intranasally vaccinated animals had significantly better viremic control, and protection from lethal infection compared to intramuscularly vaccinated animals. Notably, in a novel transmission model, intranasal vaccination reduced viral transmission to naïve co-housed mice compared to intramuscular vaccination.

**Discussion:**

Our data provide convincing evidence for the use of intranasal vaccination in protecting against SARS-CoV-2 infection and transmission.

## 1 Introduction

Since November 2019, severe acute respiratory syndrome-coronavirus-2 (SARS-CoV-2), has caused the current global pandemic and can present with a multitude of symptoms in infected individuals, such as fever, cough, general fatigue, and dyspnea, which can result in severe pneumonia or acute respiratory failure (1, 2). SARS-CoV-2 is a *Betacoronavirus* that infects humans via respiratory droplets and aerosols, resulting in a high level of virus transmission and a range of diseases (3–5). The populations at highest risk for severe COVID-19 are also those in which it has been historically the most difficult to generate protective responses following vaccination. This includes the elderly, the immunocompromised, and those with underlying health conditions, including metabolic syndrome (e.g., higher BMI, diabetes, and cardiovascular disease) (6–10). The need to develop vaccination strategies that protect against SARS-CoV-2 infection and transmission continues to be a critical question that requires continued investigation.

**Figure 1:**
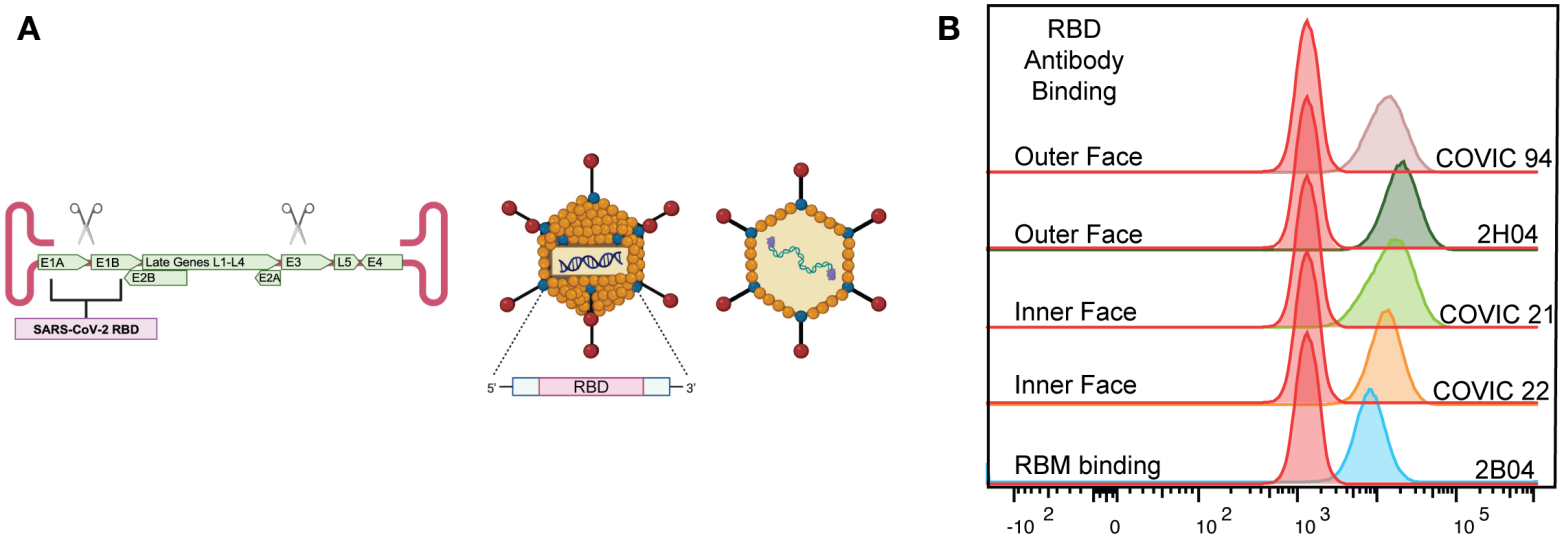
Characterization of the AdCOVID vaccine. **(A)** Representative diagram of E1/E3-deleted Ad5 vaccine encoding residues 302-543 of the receptor binding domain (RBD) of SARS-CoV-2 Wuhan1 (accession number QHD43416). **(B)** Representative flow cytometry plots showing the binding of neutralizing antibodies to AdCOVID-expressed RBD demonstrating the expression of conformational epitopes.

To enter permissive cells for replication, SARS-CoV-2 must attach to the human angiotensin-converting enzyme-2 (hACE2) receptor via the receptor binding domain (RBD) of the SARS-CoV-2 spike protein (11, 12). The RBD, located within the S1 subunit, presents an immunogenic target for neutralizing antibodies (13–15). To date, there are four known core RBD epitope classes recognized by neutralizing antibodies (**Figure 1A**) (16–18). These epitopes are critical targets for all current vaccines (19, 20). These epitopes are topologically classified into three specific motifs (receptor-binding motif, outer face of the RBD, and inner face of the RBD) based on the unique properties these regions possess (16). Antibody-mediated binding of these motifs causes neutralization by three mechanisms: competing with the hACE2 receptor, crosslinking of the RBD across spike proteins, or preventing movement of the RBD within the spike protein (13, 16).

All currently administered SARS-CoV-2 vaccines are delivered by intramuscular (IM) injection. The most widely administered SARS-CoV-2 vaccines, Pfizer/BioNTech’s Comirnaty (BNT162b2) and Moderna’s Spikevax (mRNA-1273), are mRNA platform-based vaccines that have proven to be safe and efficacious (21–26), generating protective immunity against SARS-CoV-2 Wuhan-hu-1 and several variants (27–30). Similar results have been reported in viral-vectored vaccines (31, 32). However, the durability of protection has become a concern (33, 34), as highlighted by the rapid decline in neutralizing antibody titers (35, 36). Moreover, data suggests that IM delivery does not elicit long-lasting, sterilizing immunity within the respiratory tract (37, 38). Prior experiments leveraging a protein-subunit influenza vaccine, or modified viral vaccines including modified vaccinia virus Ankara, ChAdOx-1, and parainfluenza virus type 5 have shown that intranasal (IN) vaccine delivery elicits affinity matured antibody responses in the lungs of vaccinated animals, yielding potently neutralizing IgA antibody levels. These mucosal antibodies were significantly increased compared to animals receiving conventional systemic vaccination via intramuscular immunization (39–43). Therefore, a mucosal vaccine administration route may lead to improved protection against and reduced transmission of respiratory pathogens such as SARS-CoV-2.

The continued evolution of SARS-CoV-2—combined with a lack of sterilizing immunity—has allowed a sustained cycle of transmission even with a high level of immunity within the human population. Experimental models of transmission using hamsters, ferrets, and deer mice have been developed to investigate the transmissibility of viral variants (44–46). These three models, however, lack the reagents or potential for genetic approaches to understand the role of vaccine-induced immunity in controlling transmission. With continued breakthrough infections in vaccinated individuals, it is critical to understand how the route of vaccine administration affects transmission. Laboratory mice are one of the most tractable systems to investigate the role vaccine immunogenicity has on virus transmission.

To address the possible difference in immunogenicity, protection, and transmission between varying routes of vaccine administration, we vaccinated cohorts of mice IM or IN with AdCOVID, an adenovirus serotype 5-vectored (Ad5) vaccine expressing the RBD of SARS-CoV-2. We demonstrated that the AdCOVID vaccine provides protection from a lethal SARS-CoV-2 challenge. Upon IN vaccine administration, AdCOVID primed responses capable of reducing viral replication in the lungs and brain of challenged mice. We also show that when compared to IM vaccination, IN vaccination elicits robust mucosal immunity in the lungs and limits transmission to naïve mice. Overall, our findings highlight the potential of IN vaccination to protect against the SARS-CoV-2 virus and its potential to further limit transmission using the AdCOVID platform. In addition, we report a novel model of SARS-CoV-2 transmission utilizing K18-hACE2^+/+^ transgenic mice on a C57BL/6 background, which are significantly more susceptible to mortality and have increased viral loads, permitting researchers to leverage the tractability of existing genetically modified B6 mouse lines. These studies are significant as they highlight the protective capacity of mucosal vaccination and provide a model for elucidating the immunologic determinants of SARS-CoV-2 transmission.

## 2 Materials and methods

### 2.1 Ethics statement

All animal studies were conducted in accordance with the Guide for Care and Use of Laboratory Animals of the National Institutes of Health and approved by the Saint Louis University Animal Care and Use Committee (IACUC; protocol 2771).

### 2.2 Cells and virus

Vero-human ACE2^+^ TMPRSS2^+^ (VRC, NIAID) were cultured at 37°CC in Dulbecco’s Modified Eagle Medium (DMEM) supplemented with 10% fetal bovine serum (FBS), 10mM HEPES (pH 7.3), and 10 mg/mL of puromycin antibiotic.

SARS-CoV-2 (strain USA-AZ1/2020) was obtained from BEI Resources (NR-52383) and grown on Vero human ACE2^+^ TMPRSS2^+^. The virus stocks were subjected to next-generation sequencing, and the S protein sequences were the same as the published sequence for this isolate. The virus was passaged once in Vero-human ACE2^+^ TMPRRS2 over-expressing cells and titrated by focus-forming assay (FFA) on Vero-human ACE2^+^ TMPRRS2 cells, as described previously (47, 48). All virus experiments were completed in an Animal Biosafety Level 3 (ABSL-3) facility.

### 2.3 AdCOVID vaccine

A codon-optimized SARS-CoV-2 Wuhan strain receptor binding domain (residues 302-543) was expressed by a replication-deficient human adenovirus 5 (hAd5) as previously described (49). In brief, the hAd5 vector lacks the essential E1 gene, rendering it replication-deficient. The deletion of the E3 genes allows for additional genomic space for the transgene cassette. The expression cassette is a cytomegalovirus immediate early promoter-driven transgene encoding a tissue plasminogen activator signal sequence followed by a human-codon optimized SARS-CoV-2 RBD. The stock was grown on E1-complementing PER.C6 cells and the titer was measured on HEK293 cells.

Using a panel of well-defined SARS-CoV-2 RBD mAbs, 2H04, 2B04, COVIC 21, 22, and 23, we used mAb binding to confirm the proper folding of vaccine-generated RBD. 293FT-hACE2^+^ cells were infected with AdCOVID at a multiplicity of infection of 5 for 48 hours, harvested, fixed, and permeabilized the cells, then stained with a panel of mAbs. These antibodies were specific for the functionally defined antibody epitopes established by the Coronavirus Immunotherapeutic Consortium (16).

### 2.4 Mice and infections

Transgenic K18-hACE2^+/–^ mice were purchased from Jackson Laboratories (stock:034860) and maintained as a colony in a pathogen-free mouse facility at Saint Louis University School of Medicine. The generation of K18-hACE2^+/+^ mice was completed by crossing two K18-hACE2^+/–^ mice and screening for homozygous K18-hACE2^+/+^ (Transnetyx T1371450). Six-week-old K18-hACE2 transgenic mice were immunized with 6x10^8^ infectious units (IFU) of AdCOVID in A195 buffer and administered via intramuscular (IM; hind leg) or intranasal (IN) route (25 μL per nostril per mouse, 50 μL total). All vaccinations and subsequent virus inoculations were performed under anesthesia, a ketamine/xylazine cocktail was administered intraperitoneal (IP), and all efforts were made to reduce animal suffering.

### 2.5 RT-qPCR

The viral burden for SARS-CoV-2 was measured by RT-qPCR using the following primer and probe sets from Integrated DNA Technologies (IDT) with the following sequences specific to the nucleocapsid protein: Forward 5’ GAC CCC AAA ATC AGC GAA AT 3’, Reverse 5’ TCT GGT TAC TGC CAG TTG AAT CTG 3’, Probe 5’ ACC CCG CAT TAC GTT TGG TGG ACC 3’. Synthesized SARS-CoV-2 RNA was used as a copy number control (available from IDT) to quantify the number of SARS-CoV-2 molecules present in each sample.

### 2.6 T cell stimulation

For anti-CD3 stimulation of peripheral blood lymphocytes, blood was collected via submandibular cheek bleed directly into an alkaline lysis buffer. After red blood cell lysis, cells were washed twice with complete RPMI media (10% FBS, 1X HEPES, and 1X beta-mercaptoethanol) and resuspended in complete RPMI and stimulated for 6 hours at 5% CO_2_ and 37°C in the presence of 10 μg/ml brefeldin A with 5 μg/ml of anti-CD3 (clone 2C11) or control mAb as a negative control. For peptide stimulation of splenocytes, spleens were harvested into complete RPMI medium from mice 5 days post SARS-CoV-2 boost. Single-cell suspensions were generated then 5x10^5^ cells were plated per well in a 96-well round bottom plate and stimulated for 6 hours at 5% CO_2_ and 37°C in the presence of 10 μg/ml brefeldin A and 50 μg/ml of each peptide or peptide pools. As negative controls, cells were stimulated with a pool of ZIKV envelope peptides or vehicle DMSO. For peptide stimulation of lung cells, lungs were harvested from mice, cut up into smaller pieces, and placed into a digestion buffer (final concentrations: 1mg/ml collagenase, 25 units/ml DNase I). Following digestion, single-cell suspensions were generated after cells were passed over a 100um filter and then washed with RPMI. Approximately 2x10^6^ cells were plated per well in a 96-well round bottom plate and stimulated for 6 hours at 5% CO_2_ and 37C in the presence of 10 μg/mL brefeldin A and 50 ug/mL of spike-513 peptide. As negative controls, cells were stimulated with a pool of ZIKV envelope peptides or vehicle DMSO.

### 2.7 Flow cytometry

Following stimulation of lymphocytes, cells were washed once with PBS and stained overnight in PBS at 4°C for the following surface antigens: CD4 (clone RM-4-5), CD8α (clone 53-6.7), and CD19 (clone 1D3). Cells were washed in PBS, then fixed in 2% paraformaldehyde at 4°C for 10 minutes. After fixation, cells were permeabilized with 0.5% saponin and stained in 0.5% saponin at 4°C for 1 hour for the following intracellular antigens: TNFα (clone Mab11) and IFN-γ (clone B27). After intracellular staining, cells were washed with 0.5% saponin followed by PBS. The cells were analyzed by flow cytometry using an Attune NxT focusing flow cytometer. For analysis, CD4^+^ T cells were gated on lymphocytes, CD19 negative, CD4 positive, and CD8 negative cells. CD8^+^ T cells were gated on lymphocytes, CD19 negative, CD4 negative, and CD8 positive. Antigen-specific cells were then identified as producing IFN-γ and/or TNFα at greater than 2-fold over cells stimulated with vehicle control.

For T follicular helper staining, the splenocytes and lung cells were washed with PBS and stained for CXCR5, CD62L, CD8, CD4, PD-1, CD3, and B220 in PBS overnight at 4C before being washed with PBS and run on an Attune focusing flow cytometer. T_fh_ cells were defined as lymphocytes based on forward and side scatter, singlets, B220 negative, CD3 positive, CD4 positive, PD-1, and CXCR5 high.

For plasmablast (PB) staining, the splenocytes and lung cells were washed with PBS and stained for IgM, CD19, CD138, SCA-1, CD3, and IgG in PBS and for germinal center B cells (GCB), the splenocytes and lung cells were washed with PBS and stained for GL7, CD19, IgD, CD95, CD3, and CD86 in PBS overnight at 4C. Both assays were washed with PBS and run on an Attune focusing flow cytometer. PB and GCB cells were defined as lymphocytes based on forward and side scatter, CD3 negative, CD19 positive, CD138 and SCA-1 high, and CD3 negative, CD19 positive, IgD negative, and CD95 and GL7 high, respectively.

### 2.8 SARS-CoV-2 Receptor binding domain ELISA

To determine the binding potential of polyclonal sera from SARS-CoV-2 infected mice to the receptor binding domain (RBD) of SARS-CoV-2, maxisorp ELISA plates were coated overnight at 4°C with 1 μg/ml of recombinant SARS-CoV-2 RBD protein in carbonate buffer. The following day, the plates were blocked with PBS, 5% BSA, and 0.5% Tween for 2 hours at room temperature prior to being washed. Serum from each mouse was serially diluted and added to each well and allowed to incubate for 1 hour at room temperature prior to being washed. Horseradish peroxidase conjugated goat-anti-mouse IgG secondary antibody was added and allowed to incubate for 1 hour at room temperature prior to being washed. TMB-enhanced substrate was added and allowed to incubate in the dark at room temperature for 15 minutes prior to quenching with 1N HCl. Following quenching, the absorbance of the plate was read at 450 nanometers using a BioTek Epoch plate reader.

To determine the binding potential of the antibody present in the BAL the same procedure as above was used. The measurement of the IgA responses present within the BAL after antibody binding was quantified using goat-anti-mouse IgA-HRP, and developed as described above.

### 2.9 Focus forming assay

Organs harvested from vaccinated and unvaccinated then subsequently infected mice were placed in DMEM supplemented with 5% FBS, 1X HEPES, 1X sodium pyruvate, and 1X non-essential amino acids in an O-ring tube containing a metal bead. The organs were homogenized and spun at 12,000 x g for 10 minutes at room temperature. Organ homogenate was diluted 10-fold in a 96-well round bottom plate containing DMEM as described. The homogenate was then serially diluted 10-fold and added to a monolayer of Vero-ACE2^+^ TMPRSS2^+^ cells that were plated 24 hours prior in a separate 96-well flat bottom plate. The plates were then incubated at 37C, 5% CO_2_ for 1 hour to allow attachment and entry of the virus into the cells, then overlaid with 2% (w/v) methylcellulose in DMEM supplemented with 5% FBS overnight at 37C, 5% CO_2_. After incubation, the cells were fixed using 5% paraformaldehyde for 15 minutes, then 5% formalin wash for 15 minutes before being washed with 1X PBS. The plates were washed using focus forming assay (FFA) wash buffer, before adding polyclonal anti-SARS-CoV-2 guinea pig primary antibody (BEI Cat# NR-0361) in FFA staining buffer for overnight incubation at 4C. The cells were washed with FFA wash buffer, then placed in HRP-conjugated goat anti-guinea pig IgG secondary antibody (Sigma, A-7289) in FFA staining buffer at room temperature for 3 hours. After incubation with a secondary antibody, the cells were washed with FFA wash buffer before TrueBlue peroxidase substrate (KPL) was used to stain and develop the plates before counting the foci on a BioSpot analyzer (Cellular Technology Limited).

### 2.10 Focus reduction neutralization assay

The FRNT was completed as previously described (48). Briefly, serum from each mouse was serially diluted in DMEM containing 5% FBS and combined with ~100 focus forming units (FFU) of SARS-CoV-2 and allowed to complex at 37°C and 5% CO_2_ for 1 hour in a 96-well round bottom plate. The antibody-virus complex was then added to each well of a 96-well flat bottom plate containing a monolayer of Vero-hACE2+ TMPRSS2+ cells. Following 1 hour of incubation at 37°C and 5% CO_2_, the cells were overlaid with 2% methylcellulose and returned to the incubator. After 24 hours of infection, the cells were fixed with 5% electron microscopy grade paraformaldehyde in PBS for 15 minutes at room temperature. The cells adherent to the plate were then rinsed with PBS and permeabilized with 0.05% Triton-X in PBS. Foci of infected Vero cells were stained with anti-SARS polyclonal guinea pig sera (BEI) overnight at 4°C and washed 3 times with 0.05% Triton-X in PBS. Cells were then stained with horseradish peroxidase conjugated goat anti-guinea pig IgG for 2 hours a room temperature. Cells were washed again with 0.05% Triton-X in PBS prior to the addition of TrueBlue KPL peroxidase substrate, which allows the visualization of infected foci as blue spots. The foci were visualized and counted using an ImmunoSpot CTL Elispot plate reader.

### 2.11 Histopathology of lung tissue

At six days post-infection organs were harvested into 4% paraformaldehyde solution prior to embedding in paraffin blocks for sectioning. Lungs were harvested into a 4% paraformaldehyde solution prior to embedding in paraffin blocks for sectioning. Sections were mounted, processed, and stained with hematoxylin and eosin (H&E) to observe lesions. A licensed pathologist analyzed the slides in a blinded manner for signs of inflammation and tissue damage.

### 2.12 Statistical analysis

Statistical analyses were performed using Graph Pad Prism. Statistical significance involving serology (AUC analysis and NT_50_ analysis), T_fh_ analysis, T effector analysis, and viral titer analysis was determined by the Mann–Whitney test. Weight loss was compared by one-way ANOVA, and survival was measured by the log-rank test.

## 3 Results

### 3.1 Development and antigenic testing of the AdCOVID RBD vaccine

AdCOVID is an Ad5 vectored vaccine that encodes a codon-optimized secreted form of the SARS-CoV-2 RBD from the Wuhan-Hu-1 strain as the vaccine antigen (49). Deletion of the Ad5 E1- and E3- genes prevents replication of the vector in nonpermissive cells and allows for the insertion of the gene encoding the SARS-CoV-2 RBD (**Figure 1A**). We began by investigating the conformation of the RBD antigen produced by AdCOVID. Using a panel of well-defined SARS-CoV-2 RBD monoclonal antibodies (mAbs), we sought to determine whether the AdCOVID RBD expressed the conformational epitopes that drive the development of strongly neutralizing antibodies (50–53). To interrogate the RBD, we infected 293FT-hACE2^+^ cells with AdCOVID at a multiplicity of infection of 5 for 48 hours, harvested, fixed, and permeabilized the cells, and then stained them with a panel of mAbs. These antibodies were specific for the functionally defined antibody epitopes established by the Coronavirus Immunotherapeutic Consortium (16). All RBD-specific mAbs were able to recognize the RBD generated by the AdCOVID vaccine (**Figure 1B**). This confirms that the RBD expressed by the AdCOVID vaccine exhibits the same conformational epitopes as SARS-CoV-2 RBD, which is recognized and targeted by neutralizing antibodies (13–16, 18, 54).

### 3.2 Protection phenotype of AdCOVID vaccinated mice against SARS-CoV-2 AZ1 challenge in K18-hACE2^+/-^ mice

**Figure 2:**
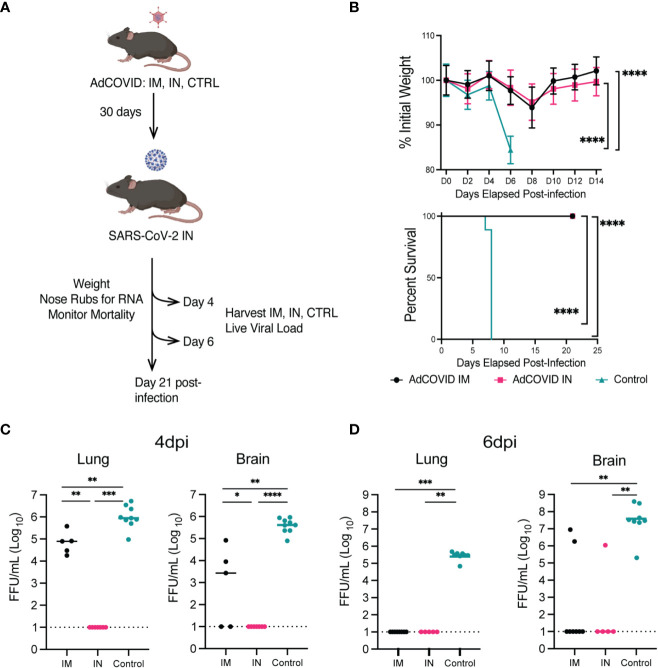
Protection phenotype: survival curve, weight-loss, and viral load of vaccinated (IM or IN) or unvaccinated (control treated) K18-hACE2^+/-^ transgenic mice. **(A)** Experimental scheme for evaluating the impact of route of vaccination on animal survival, weight loss, viral load, and disease pathology. **(B)** K18-hACE2^+/-^ mice were challenged with 2.5x10^6^ FFU of SARS-CoV-2 AZ1, and weight loss (mean +/- SEM) and survival were monitored. Weight loss was compared by one-way ANOVA, and survival was measured by the log-rank test. AdCOVID IM and IN each had significantly less weight loss than control. (**** p<0.0001) **(C, D)** Summary of infectious virus load from vaccinated and control K18-hACE2^+/-^ mice by FFA. (**** p<0.0001, *** p<0.001, ** p<0.01, * p<0.05, ns p>0.05 by Mann-Whitney test).

Based on our prior studies with SARS-CoV-2 (49), we established an experimental scheme to assess the protective efficacy of AdCOVID delivered via an IM or IN route (**Figure 2A**). In this approach, heterozygous K18-hACE2 mice (K18-hACE2^+/-^) were vaccinated once with 6x10^8^ infectious units (IFU) of AdCOVID via the IM or IN route. The control mice received PBS via the IN route. Thirty days after vaccination, mice were screened for seroconversion to confirm vaccination. The seroconverted vaccinated and control mice were then infected with a lethal challenge (2.5x10^6^ FFU) of the SARS-CoV-2 AZ1 strain via the IN route. To determine the protective efficacy of the different routes of vaccine administration, mice were either monitored for weight and survival for 14 days post-infection (dpi) (**Figure 2B**), harvested 4 or 6 dpi or to measure viremia and viral load in vaccinated and control animals (**Figures 2C, D**).

To first test the protective efficacy of the different routes of vaccine administration, K18-hACE2^+/-^ mice were vaccinated with 6x10^8^ IFU of AdCOVID via IM or IN route and screened for seroconversion after 30 days prior to a lethal challenge with SARS-CoV-2 AZ1 (IN) (**Figure 2A**). Cohorts of animals (n=10 M/F per cohort) were monitored for weight loss and mortality for 14 days post-SARS-CoV-2 infection. Unvaccinated (PBS control) K18-hACE2^+/-^ mice began to exhibit significant weight loss at 6 dpi (< 10% body weight) (**Figure 2B**). Body weights for both vaccinated groups (IM and IN) began to decline at 4 dpi, decreasing until 8 dpi before gradually regaining weight. All surviving mice fully recovered weight by 14 dpi (**Figure 2B**). Importantly, compared to the unvaccinated control, both cohorts of vaccinated mice lost significantly less weight as measured at 6 dpi (p<0.0001). Unvaccinated control K18-hACE2^+/-^ mice began to succumb to infection beginning at 7 dpi, leading to a 100% mortality by 8 dpi (**Figure 2B**). K18-hACE2^+/-^ mice vaccinated with AdCOVID either via IN or IM route were completely protected from lethal challenge, with 100% survival. Based on these survival studies, we concluded that the administration of the AdCOVID vaccine by both the IN and the IM routes protected against a lethal SARS-CoV-2 challenge.

To understand the impact of the vaccine route on viral replication, we measured viral load within several tissues, at 4 and 6 dpi, in mice from IM-vaccinated, IN-vaccinated, and control cohorts. K18-hACE2^+/-^ mice were given PBS or vaccinated with 6x10^8^ IFU of AdCOVID via IM or IN route, screened for seroconversion, and challenged with a lethal challenge of SARS-CoV-2 AZ1 (IN) (**Figure 2A**). On 4 or 6 dpi, lungs and brains were harvested and viral load was measured on Vero cells expressing human ACE2 and TMPRSS2 by focus forming assay (FFA). At 4 dpi, no infectious virus was detected in the IN-vaccinated cohort, but infectious virus could be detected in the lungs and brains of IM-vaccinated and control mice (**Figure 2C**). Infectious virus was found in the brain of 3 out of 5 IM-vaccinated and all control mice at 4 dpi. By 6 dpi, we could no longer detect the virus in the lungs of IM-vaccinated mice (**Figure 2D**). However, for the control mice, detectible virus was maintained in all eight animals at levels significantly higher than that of the IM- and IN-vaccinated cohorts. At 6 dpi, the virus was detected in the brains of IM-vaccinated mice for just 2 out of 9 animals, whereas all eight control mice supported infectious viral in the brain at this time point. Notably, we were unable to detect infectious virus in the brains of all IN-vaccinated mice at 6 dpi. Statistical analysis revealed significant differences in both the brains and lungs of vaccinated groups as compared to the unvaccinated controls. This finding suggested that the AdCOVID vaccine administered IN or IM significantly reduced viral replication in both the lungs and the brain following SARS-CoV-2 infection at 6 dpi. This data also demonstrated that SARS-CoV-2 infection of the brain is not always lethal, as observed by others (55, 56).

Next, we evaluated AdCOVID efficacy in all mouse cohorts by measuring viral genome copies in the nasal cavity (NC). By measuring nucleocapsid (N) gene transcript levels, we observed ~10^4^ copies/mL within the NC of the control mice at 1 dpi. IN vaccination, by contrast, reduced the viral load by 13-, 2.5-, 31-, and 96- fold respectively, relative to the control cohort (p< 0.01, 0.2, 0.0001, 0.0001) (**Supplemental Figure 1**). In comparison to IM vaccination, IN vaccination significantly reduced the viral RNA load within the NC by 13-fold at 1 dpi (p< 0.01). For days 3, 5, and 7 post-infection, mice immunized via either route reduced viral load equally compared to control mice. These studies showed that IN vaccination was able to limit virus replication at earlier time points, and further reduce spread to other organs such as the brain.

### 3.3 Immunogenicity of AdCOVID vaccine administered intramuscularly versus intranasally in K18-hACE2^+/–^ transgenic mice

**Figure 3:**
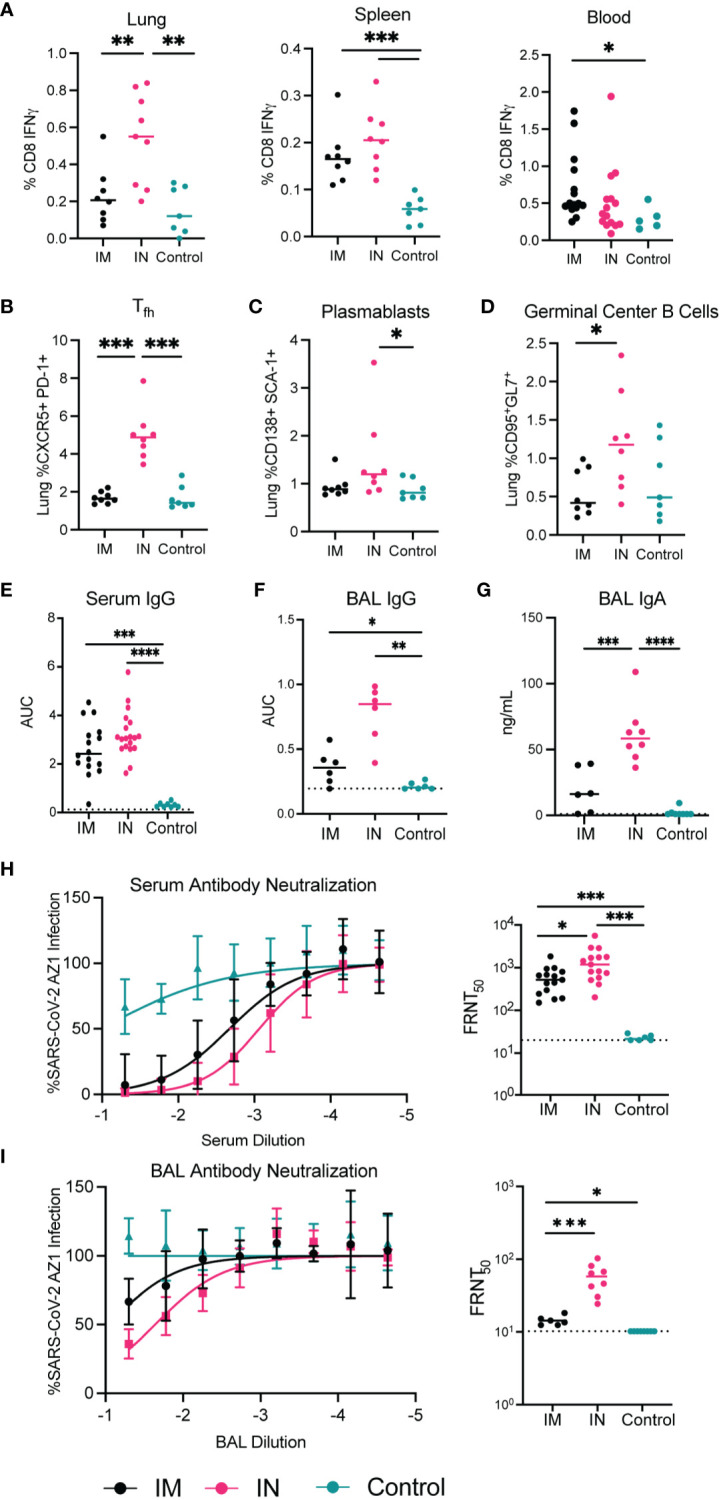
Immunogenicity of AdCOVID vaccine administered intramuscularly vs intranasally in K18-hACE2 transgenic mice 30 days post-vaccination. Seven-to-eight-week-old K18-hACE2^+/-^ mice were immunized with the AdCOVID vaccine and immunogenicity parameters were measured 30 days after vaccination. **(A–D)** Cellular responses to IM and IN vaccination to AdCOVID 30 post-vaccination. A peptide specific intracellular cytokine stimulation assay was used to quantify RBD specific CD8+ T cells in the lung, spleen, and blood **(A, B)**. Flow cytometry of infiltrating lymphocytes within the lungs was used to quantify **(B)** Tfh, **(C)** plasmablasts, **(D)** germinal center B cells. **(E, F, G)** Humoral responses in sera and BAL fluid of immunized mice were evaluated. An ELISA measured anti-RBD IgG and IgA from IM and IN vaccinated mice. **(H, I)** Neutralizing activity of sera and BAL fluid was measured by FRNT assay. **(A–G)** Mann-Whitney test was used to compare IM, to IN and control groups. (**** p<0.0001, *** p<0.001, ** p<0.01, * p<0.05.

To understand the protective immunity elicited by AdCOVID delivered either IM or IN, we evaluated the antigen-specific cellular and humoral immune responses after a single AdCOVID vaccination in the K18-hACE2^+/–^ transgenic mice. Cohorts of K18-hACE2^+/–^ mice were vaccinated with AdCOVID (6x10^8^ IFU/mouse) either IM or IN, then harvested 30 days post-vaccination for spleen, lungs, bronchoalveolar lavages (BALs), blood, and serum. Intracellular cytokine staining assays were performed on the spleen, lungs, and blood to determine the phenotype and function of antigen-specific T lymphocytes in these tissues using a sarbecovirus-specific epitope we previously identified within the RBD, spike-513 (47) (**Figure 3A**). Post-stimulation, cells were stained for flow cytometry and evaluated for the responsiveness of CD8^+^ T cells by interferon-γ (IFN-γ). Intranasal vaccination generated a significantly increased antigen-specific CD8^+^ T cell population within the lungs. There were no significant differences in CD8^+^ T cell populations within the spleen or blood after IM versus IN vaccination. This result suggests that the IN vaccination was able to specifically enhance the CD8^+^ T cell response in the lungs of the K18-hACE2^+/-^ transgenic mice.

In addition to CD8^+^ T cell cytokine production, T-follicular helper (T_fh_) cells, plasmablasts, and germinal center B cells were evaluated to determine their frequency in the lungs of IN and IM vaccinated mice compared to controls (**Figures 3B–D** **).** Similar to what was seen for the CD8^+^ T cell responses, there was a significant increase in the frequency of the T_fh_ cell and germinal center B cells in the lungs of mice vaccinated IN versus mice vaccinated IM. In data shown in **Supplemental Figure 2**, we noted that there were no differences in the percentage of plasmablasts and germinal center B cells in the spleen between the IN and IM cohorts. Importantly, while germinal center B cells were significantly elevated in the lungs following IN vaccination when compared to IM-vaccinated mice, we also noted a significant increase in the frequency of germinal center B cells found in the spleen of IN-vaccinated mice when compared to control-treated mice (**Supplemental Figure 2** ). This increase in spleen germinal center B cell populations after IN vaccination is suggestive of a systemic immune response to IN AdCOVID vaccination. Overall, this data suggests that the administration of AdCOVID via IN vaccination induces a potent cellular immune response in the lungs, thereby priming the adaptive immune response at the site of infection.

To determine if the route of administration would also alter the quantity and quality of the protective antibody response, we measured the SARS-CoV-2 specific antibody response in the lungs of IM and IN vaccinated mice as compared to controls. We quantified SARS-CoV-2 RBD-specific IgG antibodies in the serum and BAL via ELISA at 30 days post-vaccination (**Figures 3E, F**). Detection of IgG specific for RBD in the serum of vaccinated mice showed no significant differences between the two routes of vaccination (**Figure 3E** ). However, the SARS-CoV-2 specific antibody response was significantly higher in mice vaccinated IM and IN when compared to control-treated mice. We also observed a similar trend with the RBD-specific IgG antibody response within the BAL with both an IM and IN vaccination increasing IgG within the BAL significantly over control vaccination (**Figure 3F**). With our interest in understanding the role of IN vaccination, we also quantified the level of IgA specific for RBD after IN and IM vaccination within the BAL. In this case IN vaccination generated significantly higher levels of IgA antibody in comparison to both the IM and control vaccination (**Figure 3G**). These results confirm that vaccination by either route can induce a systemic SARS-CoV-2 specific antibody response, while IN vaccination can generate a more robust IgG and IgA response within the lung.

To test the neutralization capacity of the antibody response from the IN- and IM-vaccinated mice we performed focus-reduction neutralization tests (FRNTs). The FRNTs were done on both the serum and BAL fluid (**Figures 3H, I**). The serum isolated from IN-vaccinated mice showed significantly higher neutralizing activity against SARS-CoV-2 AZ1 than IM-vaccinated mice (**Figure 3H**). The neutralizing activity of the BAL fluid similarly showed higher neutralizing activity in mice immunized IN compared to IM (**Figure 3I**). This increase in neutralizing antibody titer demonstrates one advantage of IN vaccination for respiratory pathogens such as SARS-CoV-2.

Overall, in evaluating the immunogenicity of AdCOVID, we showed that IN vaccination stimulates cytokine production by CD8^+^ T cells in the lungs, a more robust T_fh_ response, and a subsequent germinal center B cell response. This translated to high titers of IgG antibodies in bronchoalveolar lavage fluid and serum, higher levels of IgA within the BAL, and produced neutralizing antibodies in both the periphery and BAL. This indicates that AdCOVID stimulates a humoral and cellular response, and specifically when administered intranasally provides higher titers of antibodies and neutralizing activity when compared to mice vaccinated intramuscularly. These immunological parameters provide a potential mechanism for the control of virus load within the lower respiratory tract.

### 3.4 Histological analysis of AdCOVID vaccine administered intramuscularly versus intranasally in SARS-CoV-2 challenged K18-hACE2^+/–^ transgenic mice

**Figure 4:**
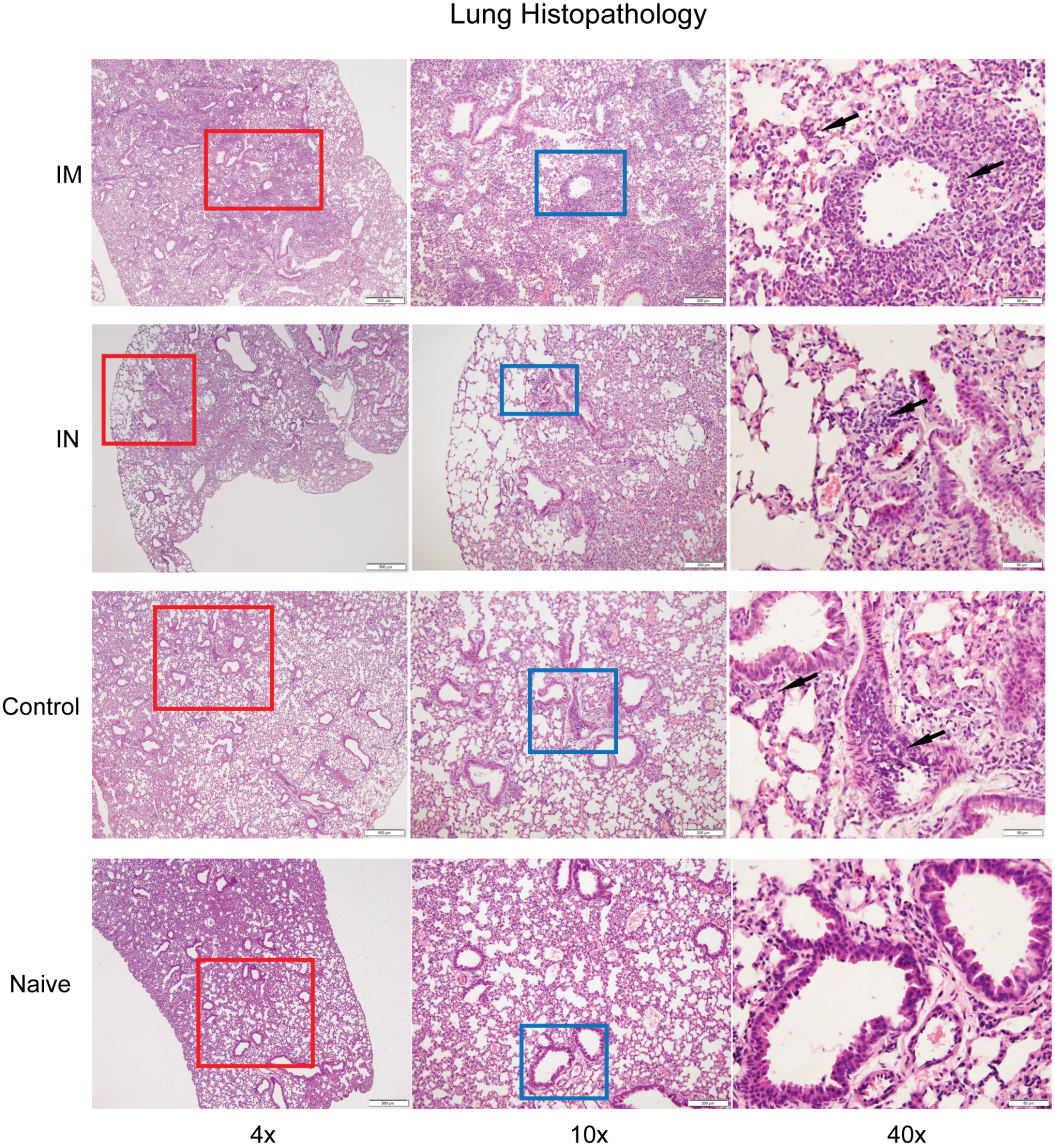
Histopathological analysis of SARS-CoV-2 infection after vaccination. Hematoxylin-and -eosin staining of lung sections from uninfected and infected K18-hACE2^+/-^ mice six days post-infection. The lungs from control or IM vaccination show perivascular, intravascular, and intraseptal inflammation (black arrows), while IN-vaccinated challenged mice show small foci of inflammation in comparison to a naïve lung. Images are at 4x (column 1), 10x (column 2), and 40x (column 3).

Based on the immunogenicity studies, we wanted to assess the extent of inflammation and infiltration of immune cell populations in our vaccinated and challenged animals. This was of particular interest as we were unable to detect replicating virus in the lungs of the IN-vaccinated animals (**Figure 2C**) but did observe some evidence of infection as suggested by the weight loss data observed between days 4 and 10 (**Figure 2B**). To determine the extent of inflammation, histopathological analysis was performed on the lungs of IN- and IM-vaccinated mice, as well as PBS control mice, at 6 dpi, with a lung section from an uninfected K18-hACE2^+/–^ shown for comparison. Control mouse lungs showed intravascular, perivascular (both venous and arteriolar), and rare peribronchiolar cellular infiltration, indicative of SARS-CoV-2-induced inflammation (**Figure 4**). IM-vaccinated mice showed venous, arteriolar, and intraseptal mononuclear inflammation, as well as significant cellular infiltrate in the lung bronchioles. Despite the multiple foci found in the lungs of control and IM-vaccinated mice, IN-vaccinated mice showed a reduction in pathology in the lungs. The lungs of IN-vaccinated mice showed cell infiltration which presented as small, scattered foci around the bronchioles and the veins (**Figure 4** **).** While this was indicative of disease, the extent of these pathological findings was less than that found in IM-vaccinated or control mice. IN administration of AdCOVID reduced inflammation in the lungs caused by SARS-CoV-2 infection and lessened disease severity, ultimately suggesting increased protective capacity against lethality, morbidity, and lung pathology.

### 3.5 Development of K18-hACE2^+/+^ transgenic mice as a stringent challenge model of intramuscular versus intranasal AdCOVID vaccination

Our studies thus far have demonstrated that the IM and IN routes of vaccination were both able to protect susceptible mice from a lethal challenge. However, the IN route of vaccine administration afforded better control of viral replication and the capacity to reduce pathology as compared to IM vaccination. Furthermore, we showed that IN vaccination induced significantly stronger immune responses. Based on these results, we sought an *in vivo* model where we could more stringently challenge our hypothesis that IN vaccination would provide better protection against SARS-CoV-2.

The level of human ACE2 receptor expression is one potential factor that can account for variation in vaccine-mediated protection and disease progression (57, 58). Studies have demonstrated that expression of ACE2 correlates with viral infectivity (59, 60). To develop an animal model that was more susceptible to SARS-CoV-2, we bred heterozygous K18-hACE2^+/–^ transgene mice together and selected for homozygous K18-hACE2^+/+^ transgene expression (**Supplemental Figure 3A**). To determine if homozygous mice expressed higher levels of the hACE2 gene than heterozygous K18-hACE2 mice, we harvested the brain and lungs and quantified the expression of the hACE2 receptor in these two organs via RT-qPCR (**Supplemental Figure 3B**). We identified a significant increase in the expression of the hACE2 transgene in homozygous K18-hACE2^+/+^ mice compared to heterozygous mice at the transcriptomic level.

**Figure 5:**
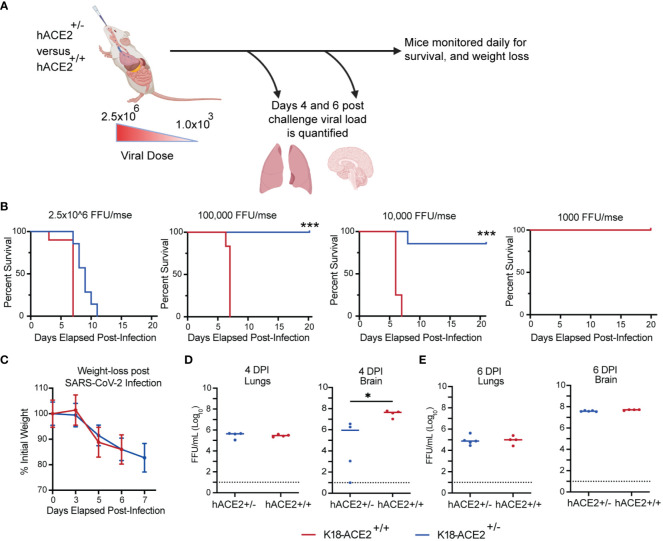
Impact of hACE2 expression level on the susceptibility of K18-hACE2 transgenic mice. **(A)** Experimental scheme for evaluating the impact of hACE2 expression on animal survival, weight loss, and viral load. **(B)** K18-hACE2 ^+/-, +/+^ mice were challenged with decreasing doses of SARS-CoV-2 AZ1 starting at 2x10^6^ FFU and survival was monitored. **(C)** Body weight change over time and **(D, E)** infectious viral load in the lungs and brain were measured on days 4 and 6 post-infection. Weight change data are the mean +/- SEM comparing K18-hACE2^+/+, +/-^ groups. Viral levels were measured by FFA. Points show median values +/- Std Dev. (*** p<0.001, * p<0.05, ns p>0.05).

To validate the K18-hACE2 homozygous mouse model for use in SARS-CoV-2 infection experiments, we determined the sensitivity and susceptibility to severe disease by infecting mice with decreasing doses of SARS-CoV-2 (**Figure 5A**). We infected K18-hACE2 homozygous and heterozygous mice via IN challenge with four doses of SARS-CoV-2 AZ1 strain: 2.5x10^6^ FFU, 1x10^5^, 1x10^4^, or 1x10^3^ FFU (**Figure 5B**). Mice from both cohorts succumbed to SARS-CoV-2 infection when given 2.5x10^6^ FFU intranasally. However, we found significant differences in survival between the homozygous and heterozygous mouse cohorts when we continued to serially dilute the virus. All homozygous K18-hACE2 mice succumbed to SARS-CoV-2 infection after challenge with the 50- and 500-fold lower viral challenges. When both K18-hACE2 homozygous and heterozygous mice were infected IN with 2.5x10^6^ FFU AZ1 strain, we observed a similar rate of weight loss (**Figure 5C**) and similar infectious viral load within the lungs at days 4 and 6 (**Figures 5D, E**). However, in K18-hACE2 homozygous mice we observed an earlier entry of SARS-CoV-2 into the central nervous system, resulting in a ~30-fold higher level of virus on day 4, but by day 6 the levels of infectious virus between homozygous and heterozygous mice were similar. We observed similar kinetics and distribution when the viral genome copy number was analyzed (**Supplemental Figure 3C**). Overall, the homozygous mouse model showed increased susceptibility to SARS-CoV-2 disease, making it an ideal model for more stringent vaccine testing.

**Figure 6:**
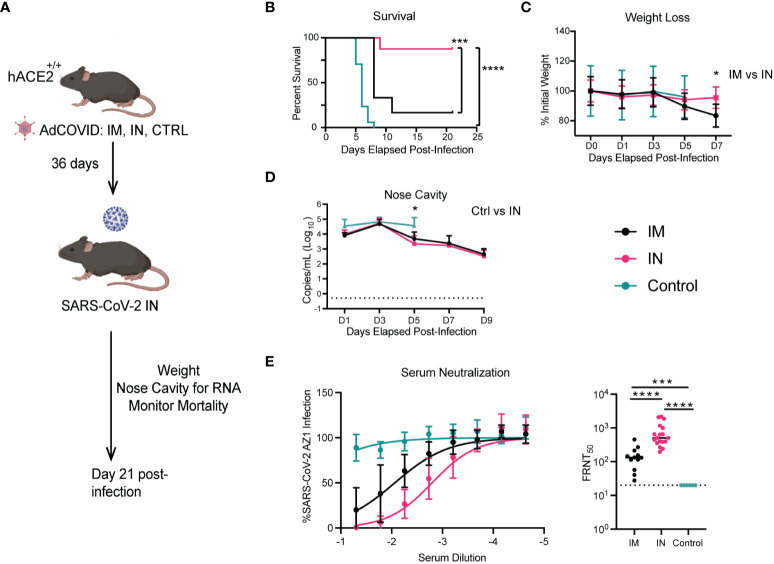
Impact of vaccination route on the protection of K18-hACE2^+/+^ transgenic mice. **(A)**. Experimental scheme for evaluating the route of vaccination on animal survival, weight loss, and viral load. **(B)**. At 36 days post-vaccination, mice were challenged with 2.5x10^6^ FFU of SARS-CoV-2 AZ1. Animal survival was measured for 21 days. **(C)**. Body weight change over time and **(D)** viral RNA levels in the NC were measured every other day for 9 days. Weight change data are the mean +/- SEM comparing vaccine to control groups. Viral RNA levels were measured by RT-qPCR. Points show median values +/- Std Dev. **(E)** Neutralizing activity of serum antibody of homozygous K18-hACE2^+/+^ mice immunized IM, IN, or control were evaluated by FRNT assay. (**** p<0.0001, *** p<0.001, * p<0.05). Weight loss was compared by one-way ANOVA, survival was measured by the log-rank test, and viral RNA level and neutralizing antibody were compared by the Mann-Whitney test.

Using the homozygous K18-hACE2 mice, we were then able to compare the protective capacity of the IN versus the IM route in a more stringent challenge model. To this end, we vaccinated three cohorts of K18-hACE2 homozygous mice via the IM or IN route with 6x10^8^ IFU of AdCOVID, and a negative control cohort (**Figure 6A**). After six weeks, vaccinated and control K18-hACE2^+/+^ mice were infected with a 500x lethal dose of 2.5x10^6^ FFU of SARS-CoV-2 isolate USA-AZ1/2020 via the IN route. The mice were monitored for survival (**Figure 6B**), weight loss (**Figure 6C**), and viral genome copy number in the nasal cavity (**Figure 6D**). Negative control homozygous K18-hACE2 mice began to succumb to severe disease starting at 5 dpi, resulting in complete mortality by 8 dpi (**Figure 6B**). Homozygous K18-hACE2 mice that received IM vaccination began to succumb to infection at 8 dpi, ultimately leading to a 17% survival rate by day 11 for this cohort. The delayed mortality observed in the IM-vaccinated mice compared to the control resulted in a significant difference in the mean time-to-death between the PBS control and IM-vaccinated groups (p <0.002). However, most notably, IN-vaccinated mice showed considerable protection compared to IM-vaccinated mice, with a significant increase in survival post-infection with an 87% survival rate (p <0.006 IM vaccinated; p <0.0001 control mice) (**Figure 6B**). The weights of the IM and control cohorts decreased by 5 dpi, with a further reduction in weight of the IM-vaccinated cohort through 7 dpi, while the IN-vaccinated cohort showed only a slight decrease in weight on 5 dpi and a rebound in weight on day 7 (p < 0.006; IM vs IN day 7) (**Figure 6C**). However, as the IM-vaccinated mice began to succumb to infection by 8 dpi, we could no longer compare the weights between the vaccinated cohorts. In analyzing viral genome copy number within the nasal cavity, in all three cohorts of infected mice, SARS-CoV-2 viral RNA was detected at all time points up to 9 dpi (**Figure 6D**). IN vaccination led to a significant reduction of viral RNA at 5 dpi as compared to the controls. However, the viral RNA load between the IN and IM mice was similar at all time points, with both cohorts showing viral RNA load peaking at 3 dpi with a subsequent decrease by 9 dpi (**Figure 6D**).

Next, we wanted to determine if there were differences in neutralizing capabilities of the antibodies generated after IM and IN vaccination of K18-hACE2^+/+^. Using serum taken 6 weeks after vaccination with AdCOVID and prior to infection with SARS-CoV-2, we found a 5-fold (p <0.0001) increase in the neutralizing capacity of antibodies from the IN-vaccinated mice compared to the IM-vaccinated mice (**Figure 6E**). This may explain the increase in mortality experienced by the IM-vaccinated mice, where only 17% of mice from this cohort survived, compared to the 87% survival in the IN-vaccinated mice. Overall, IN vaccination with AdCOVID in K18-hACE2 homozygous mice resulted in significant increases in the neutralizing antibody and a decrease in mortality compared to the IM-vaccinated and unvaccinated cohorts.

### 3.6 Vaccine-mediated protection against SARS-CoV-2 transmission in K18-hACE2^+/+^ mice

**Figure 7:**
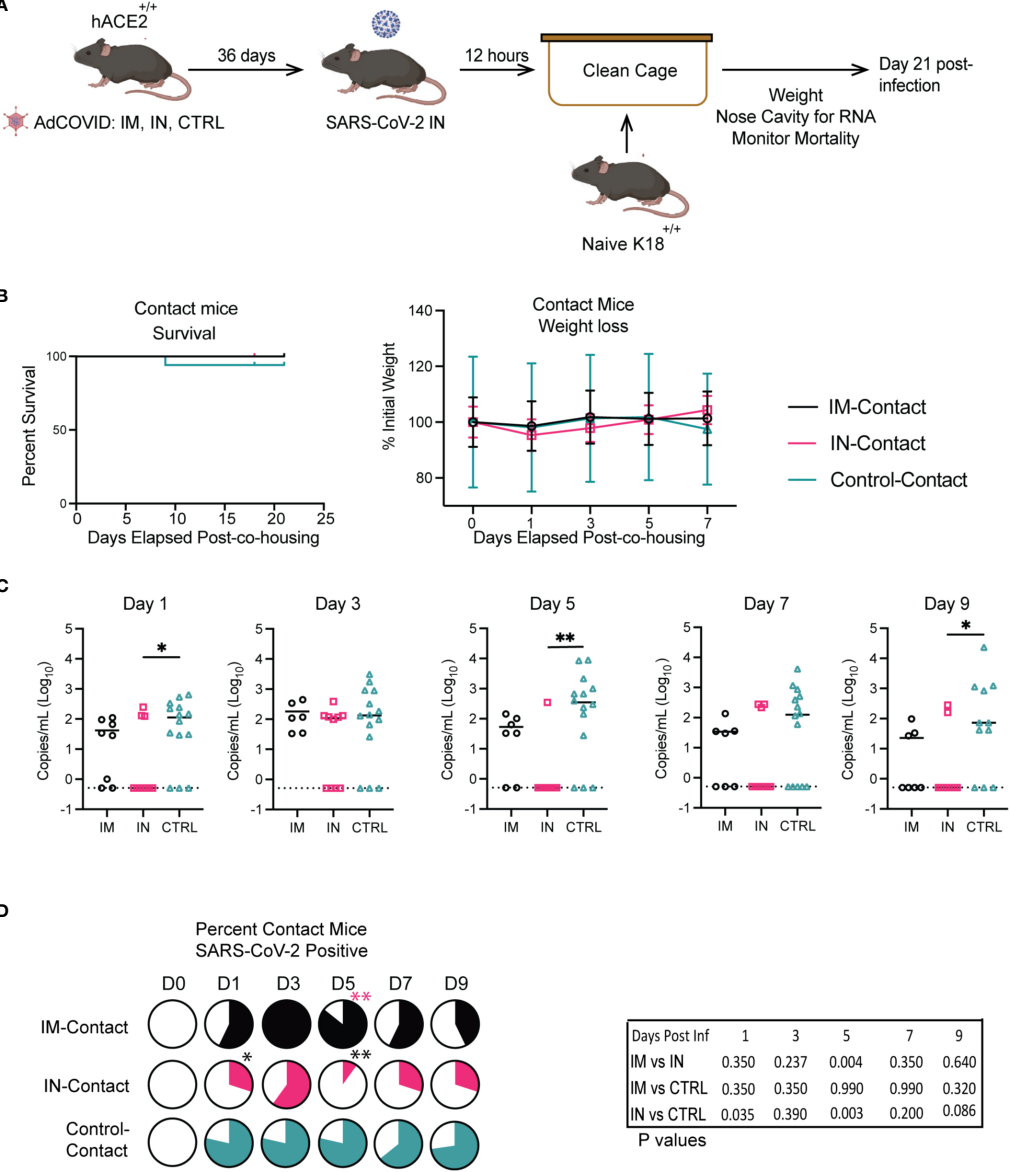
Impact of vaccination route on transmission in K18-hACE2^+/+^ transgenic mice. **(A)** Experimental scheme for measuring viral transmission from vaccinated mice to unvaccinated mice. **(B)** IM, IN, and Control-Contact, naïve mice, were monitored for animal survival for 21 days and weight loss. **(C)** Viral RNA levels in the NC were measured every other day for 9 days by RT-qPCR. (** p<0.01, * p<0.05, ns p>0.05 by Mann-Whitney test). **(D)** Percentage of IM, IN, and Control-contact animals that had detectible SARS-CoV-2 RNA in the NC measured every other day for 9 days by RT-qPCR. (** p<0.01, * p<0.05, ns p>0.05) Animal survival was measured by the log-rank test, viral RNA level was compared by the Mann-Whitney test, and the percentage of animals SARS-CoV-2+ was measured by the Fisher exact test.

Finally, using our more susceptible murine model, we next sought to investigate the potential role of vaccination in limiting transmission. From our previous studies with the heterozygous K18-hACE2 mice, we knew that vaccination via the IM route led to significantly higher viral titers in the lungs of mice as compared to the IN route, wherein mice had no detectible infectious viral titer. To test whether IN vaccination could limit transmission compared to IM vaccination, we completed similar studies to those described for the stringent challenge. In this approach, we vaccinated cohorts of homozygous K18-hACE2^+/+^ mice via the IN and IM routes (**Figure 7A**). Then, control, IN-, and IM-vaccinated mice were intranasally infected with 2.5x10^6^ FFU of SARS-CoV-2 isolate USA-AZ1/2020, and infection was allowed to progress. Twelve hours post-infection, the control, IM-, and IN-vaccinated infected cohorts were separated into groups of two mice per cage and placed into new clean cages with two naïve K18-hACE2^+/+^ mice, referred to as *control-contact*, *IM-contact, or IN-contact mice*. One day prior to co-housing with infected mice, the contact mice were administered 1 mg of anti-type I interferon receptor 1 (IFNAR1) monoclonal antibody (MAR1-5A3) (61–63). The anti-IFNAR1 MAR1-5A3 antibody was used to enhance the susceptibility of the contact mice to SARS-CoV-2 infection to allow us to detect a transmission event. Upon co-housing into clean cages both vaccinated-infected mice were allowed to directly interact with naïve K18-hACE2 homozygous mice for the rest of the experiment.

To determine vaccine efficacy and transmission of SARS-CoV-2, mortality for all cohorts was monitored for 21 days post-infection (**Figure 7B**). All contact mice were weighed prior to infection exposure and post-exposure for 21 days to identify significant changes in weight as an indicator of disease (**Figure 7B**). We observed no significant differences in weight between the contact groups. For the contact mice, there were no significant differences in survival between the direct-contact -IM, -IN, or -control cohorts. However, one control-contact mouse did succumb to SARS-CoV-2 infection.

At 14- and 21- dpi, the contact mice from each group were bled to measure IgM and IgG antibodies against SARS-CoV-2 RBD by ELISA. A significant increase in IgM antibodies was detected in the control-contact group versus the IN-contact group, and one animal from the IM-contact cohort (**Supplemental Figure 4A**). No IgM antibodies were detected in animals from the IN-contact vaccinated groups. The sera collected 21 dpi from all contact mouse cohorts was analyzed via anti-RBD ELISA for IgG antibodies to determine the number of mice whose antibody responses showed class switching upon transmission of SARS-CoV-2 after co-housing with infected mice. The majority of animals in the contact groups had detectible IgG antibodies to RBD (**Supplemental Figure 4B**). All of the IM-contact and control-contact mice developed IgG responses specific for RBD, with 6 of 9 IN-contact mice seroconverting. It is important to note that the evidence of seroconversion does not definitively indicate a transmission event, as exposure to viral antigens independent of infection can occur with co-housing.

To determine if we could detect the virus in the contact mice, all contact mice were monitored by RT-qPCR for viral genome copy number within the NC every other day for 9 dpi (**Figures 7C, D**). The IM-contact and IN-contact groups showed peak viral RNA load at 3 days post-co-housing (dpch) with decreasing viral load through day 9 (**Figure 7C**). The control-contact mice showed significantly higher viral RNA load on days 1, 5, and 9 post-co-housing in comparison to the IN-contact cohort. We next quantified the number of contact mice in each cohort that were positive for SARS-CoV-2 viral RNA in the NC (**Figure 7D**). We found that co-housing with the IN-contact cohort resulted in fewer mice presenting with detectable amounts of viral RNA in the NC when compared to the IM-contact cohort on 5 dpi. Furthermore, significantly fewer IN-contact mice tested positive for viral RNA compared to control-contact mice on 1 and 5 dpi (**Figure 7D**). These results show that IN vaccination limited transmission to unvaccinated contact mice relative to IM and control cohorts.

## 4 Discussion

An efficacious SARS-CoV-2 vaccine that stimulates durable humoral and cellular immunity has proved to be challenging, as evidenced by the subsequent emergence of variants of concern after the widespread distribution of vaccines (64–70). Due to this issue, global efforts to develop a SARS-CoV-2 vaccine that induces a robust neutralizing antibody response, memory T cells, and protection against transmission continue, with multiple vaccine strategies being used to control the current pandemic (52, 53, 71–75). Owing to the rapid waning of the antibody response observed with the first-generation COVID-19 vaccines, it is becoming evident that it is critical to further investigate the immunogenicity of IN versus IM-delivered vaccines, particularly as variants of concern continue to emerge. In response to these efforts, Altimmune developed an adenoviral-vectored vaccine encoding the RBD of the SARS-CoV-2 spike protein (AdCOVID) that conveys robust and durable immunity. Here we studied the effectiveness and protective capacity of two different routes of administration in K18-hACE2 mice. Furthermore, we developed a novel system to study vaccine-mediated transmission prevention. We show that IN vaccination with AdCOVID induces superior protective capacity and limits transmission *in vivo* from a lethal SARS-CoV-2 challenge when compared to IM vaccination.

Protection against morbidity and mortality is the hallmark of vaccination of the human population. We show that IN and IM administration of AdCOVID protects mice from mortality and significant weight loss when infected intranasally with a validated lethal dose of SARS-CoV-2 AZ1, regardless of the vaccine route of administration. Both the IM and IN routes of administration significantly reduced infectious virus titer and viral replication in both organs tested when compared to control-treated mice at both time points, as expected. However, the protection phenotype conferred by IN AdCOVID vaccination was significantly increased compared to IM delivery. IN-vaccinated mice exhibited a significant reduction in infectious virus titer and viral replication in the lungs and the brain of mice at 4- and 6- dpi as opposed to IM vaccination.

In addition to the protection phenotype conferred via IN vaccination, this route of administration induced a robust humoral and cellular immune response by generating significantly more potent neutralizing antibodies, antigenic-specific CD8^+^ T cells, and a significantly higher frequency of T_FH_ and germinal center B cells in both the lungs and spleens of K18-hACE2^+/-^ mice when compared to the IM-vaccinated cohorts. We show not only the induction of both the humoral and cellular arms of the adaptive immune system when vaccinated with AdCOVID, but also the induction of mucosal immunity when the IN route of administration was employed, as evidenced by high levels of IgG and IgA within the BAL. These results are in line with previous studies performed in mice utilizing the IN immunization route inducing mucosal immunity, specifically in B cell populations and IgA secretion (39), as well as the possibility of mucosal immunity (76–78) becoming activated faster in resident tissues (79–81) and protecting mice from variants of concern (76–78, 82). Since vaccination is undoubtedly essential to stimulate a protective humoral response, it is equally important for a vaccine to induce a cellular response that will generate antigen-specific memory T cells that will aid in the clearance of a pathogen (79–81). While induction of a cellular response via vaccination is variable (83–85), we show a sustained antigen-specific CD8+ T cell response both in the resident lung tissue and in the periphery four weeks (30 days) after mice were vaccinated intranasally with AdCOVID. It is evident that the robust, sustained immune response generated by intranasal AdCOVID vaccination provides a possible route of administration and a vaccine strategy that can be explored in further studies as a potential next-generation approach to SARS-CoV-2 vaccination.

We also found that mice vaccinated intranasally show a reduction in SARS-CoV-2 transmission to a naïve population compared to the IM-vaccinated cohort. We have demonstrated a transmission mouse model that shows successful transmission of SARS-CoV-2 from an infected cohort of mice to an uninfected cohort of mice when co-housed together. For all three direct contact cohorts for IM-vaccinated, IN-vaccinated, and unvaccinated controls, we found that a viral load exists in the NC of the mice as measured by virus genome copy number. We demonstrate seroconversion and class-switching of antibodies from IgM to IgG by 21 days post-co-housing in the direct-contact mouse cohort. This is evidence of a successful transmission model utilizing the K18-hACE2 homozygous transgenic mouse model, which is sensitive and susceptible enough to be indirectly infected with SARS-CoV-2 following type I IFN blockade via mAb. We also show that in direct-contact mice co-housed with IN-vaccinated cohorts, transmission occurred less frequently when compared to the IM-vaccinated cohorts. We also demonstrate that IN vaccination of K18-hACE2 homozygous mice with AdCOVID confers significant protection from mortality and indicators of disease upon SARS-CoV-2 challenge compared to the IM and control unvaccinated cohorts. Intranasal administration of AdCOVID induced significant neutralizing antibodies that could bind to, recognize, and neutralize SARS-CoV-2 AZ1, reducing the mortality and weight loss in these mice compared to the IM and control-unvaccinated cohorts. It is evident that the intranasal route of vaccine administration against respiratory pathogens induces significant immune responses not offered by the traditional intramuscular route, such as mucosal immunity, cellular immune response, and potent neutralizing antibodies (86, 87). Further investigation of vaccination with the intranasal route of administration with additional respiratory pathogens and the immune response is needed.

### 4.1 Limitations of the study

We show robust and efficacious vaccination using an adenoviral vector vaccine encoding the SARS-CoV-2 receptor binding domain which induces significant immune responses, a protective phenotype, and a reduction in transmission to a naïve cohort in K18-hACE2 mice when administered intranasally. The murine model used in these studies highly expresses the hACE2 receptor under the cytokeratin-18 promoter (49), whereas this protein is variably expressed in the human population and may have a role in the replication of the SARS-CoV-2 virus in susceptible and permissive cells (88–91). The differences in hACE2 expression in humans cannot be directly equated to our two K18-hACE2 murine models, producing this limitation.

Since the inception of the SARS-CoV-2 pandemic in 2020, there have been multiple variants of concern that have detrimental effects on both vaccinated and unvaccinated populations (92–96). The variants of the SARS-CoV-2 virus contain major mutations found within the spike protein, and due to the COVID-19 vaccines encoding a variation of the spike protein, mutations in these variants have since led to immune escape and evasion, higher viral transmissibility and infectivity, and lower neutralization capabilities of vaccine generated antibodies (64–70). In the present study, we focused on the abilities of the antibodies generated from the vaccine to bind to the SARS-CoV-2 receptor binding domain (Wuhan1) as well as the neutralizing capabilities against the SARS-CoV-2 AZ1 strain. We also focused our efforts on the role of ACE2 expression in vaccine-mediated protection and transmission. However, we did not determine whether these antibody binding and neutralizing capabilities in AdCOVID-vaccinated mice were consistent across the variants. Determining vaccine immunogenicity and protection within high and low ACE2 expression and the potential to limit transmission among novel SARS-CoV-2 variants is a possible future direction of research.

## Data availability statement

The datasets presented in this study can be found in online repositories. The names of the repository/repositories and accession number(s) can be found in the article/**Supplementary Material**.

## Ethics statement

The animal study was reviewed and approved by Saint Louis University IACUC.

## Author contributions

Conceptualization, AD, BG, JS, JB, and AP; Formal Analysis, AD, BG, JS, JB, and AP; Investigation, AD, EG, ES, MH, TS, KS, JZ, JB, and AP. Methodology, AD, EG, ES, MH, TS, TM, MS, BG, JS, JB, and AP. Funding Acquisition; JS, BG, AP, JB, and MR. Resources, JB, AP, JZ, BG, MR, JS; Visualization; Writing (Original Draft), AD, BG, JS, JB, and AP; Writing (Review and Editing) AD, BG, JS, JB, and AP; Project Administration, BG, JS, JB, and AP. All authors contributed to the article and approved the submitted version.
