# Supplementary material for: The role of vaccination route with an adenovirus-vectored vaccine in protection, viral control, and transmission in the SARS-CoV-2/K18-hACE2 mouse infection model
Source: Front Immunol. 2023 Aug 16;14:1188392. doi: 10.3389/fimmu.2023.1188392 (PMC10469340; doi:10.3389/fimmu.2023.1188392)
Supplement: Supplementary file 1 [file DataSheet_1.docx]

Supplementary Material

The role of vaccination route with an adenovirus-vectored vaccine in protection, viral control, and transmission in the SARS-CoV-2/K18-hACE2 mouse infection model.
